# Effects of Juhongtanke oral solution on alleviating the symptoms of community-acquired pneumonia: A multicenter, prospective, randomized controlled trial

**DOI:** 10.3389/fphar.2022.1027901

**Published:** 2022-10-20

**Authors:** Min Liang, Linhui Hu, Ning Luo, Hualiang Lv, Zhihua Chen, Jianping Mo, Meiyan Yang, Ying Lin, Chunbo Chen

**Affiliations:** ^1^ Department of Respiratory and Critical Care Medicine, Maoming People’s Hospital, Maoming, China; ^2^ Department of Critical Care Medicine, Maoming People’s Hospital, Maoming, China; ^3^ Department of Clinical Research Center, Maoming People’s Hospital, Maoming, China; ^4^ Department of Pulmonary Disease, Gaozhou Hospital of Traditional Chinese Medicine, Maoming, China; ^5^ Department of Pulmonary Disease, Maoming Traditional Chinese Medicine Hospital, Maoming, China; ^6^ Department of Respiratory and Critical Care Medicine, The First People’s Hospital of Zhaoqing, Zhaoqing, China; ^7^ Department of Intensive Care Unit of Cardiac Surgery, Guangdong Cardiovascular Institute, Guangdong Provincial People’s Hospital, Guangdong Academy of Medical Sciences, Guangzhou, China; ^8^ Department of Critical Care Medicine, Guangdong Provincial People’s Hospital, Guangdong Academy of Medical Sciences, Guangzhou, China; ^9^ Department of Emergency, Maoming People’s Hospital, Maoming, China; ^10^ The Second School of Clinical Medicine, Southern Medical University, Guangzhou, China

**Keywords:** Juhongtanke oral solution, traditional Chinese medicine, CAP, effect, remission, symptom

## Abstract

**Introduction:** The timely alleviation of symptoms is essential for managing community-acquired pneumonia (CAP). Juhongtanke oral solution is a traditional marketed Chinese patent medicine believed to ease CAP symptoms. The currently available evidence is based on a few retrospective studies of patients with various types of pneumonia, whereas robust randomized controlled trials (RCTs) that support this notion are lacking.

**Material and methods:** In this multi-center, prospective RCT, patients were randomly allocated to receive routine treatment alone or a combination of Juhongtanke oral solution (20 mL q8h) for 5 days and maintained for an additional 3-day safety observation period. The primary outcome was Breathlessness, Cough, and Sputum Scale (BCSS) score evaluated on day 5. Secondary outcomes included the evaluation of cough and dyspnea items in the Visual Analogue Scale (VAS) from days 1–5, remission rate in BCSS and VAS during the treatment course, and the length of hospitalization and in-hospital mortality.

**Results:** Of 272 patients assessed for eligibility, 240 were enrolled in the study (n =120 per group). The mean difference in BCSS evaluated on day 5 was a median 1 point [95%CI (1.00, 2.00)], significantly lower in the treatment group compared with the control group (*p* < 0.001). Similar results were observed in VAS on day 5, with statistics of a median 2 points [95%CI (1.40, 2.50)] in the cough item and a median 1 point [95%CI (0.50, 2.00)] in the dyspnea item, significantly lower in the treatment group compared with the control group (both *p* < 0.001). The treatment group had a favorable outcome in BCSS and VAS remission rate assessments compared with the control group, with 99.50% vs. 89.17% in BCSS (*p* = 0.01), 98.33% vs. 75% in the cough item of VAS (*p* < 0.001), and 88.33% vs. 62.50% in the dyspnea item of VAS (*p* < 0.001), respectively. No notable adverse effects were observed during the study. No differences were observed in the length of hospitalization between groups (with a median of 7 days for both groups, *p* = 0.871).

**Conclusion:** Juhongtanke oral solution may be considered to alleviate the clinical symptoms of CAP.

## Introduction

Community-acquired pneumonia (CAP) is infectious pneumonia that develops in individuals outside the hospital setting (Metlay et al., 2019). Despite the availability of vaccines and broad-spectrum antibiotics, CAP remains one of the major life-threatening diseases, ranking as the third leading cause of death worldwide (Collaborators GMaCoD, 1980). The economic burden also cannot be neglected. In the United States and Europe, CAP accounts for an economic loss of $8.4 billion and €46 billion per year, respectively (Niederman et al., 1998; The economic burden of lung disease, 2013). The most common symptoms of patients with CAP are cough, expectoration, and dyspnea. These complications affect the recovery and finally result in the prolonged course of the disease (Menéndez et al., 2004; Rosón et al., 2004; Aliberti et al., 2008). Consequently, adjuvant therapies that benefit symptom alleviation have been recommended by current clinical guidelines (Cao et al., 2018; Chinese Medical Association, 2019; Metlay et al., 2019). However, no consensus has been reached regarding the therapeutic agents covered by these guidelines. Several medications, such as carbetapentane, mucosolvan, and dextromethorphan, have been wildly applied to ameliorate respiratory symptoms in clinical practice. Although most of them are effective in treating a single symptom, they are ineffective in treating patients who suffer from two or more discomforts. Therefore, studies should aim to discover effective and safe medications that are antitussives, expectorants, and anti-dyspnea for patients with CAP.

Traditional Chinese medicine (TCM), which includes but is not limited to Chinese patent medicines, acupuncture, and herbs, has been applied to treat various diseases in East Asia for centuries (Lang et al., 2020; Xiao et al., 2020; Hu et al., 2021a). As a typical species of TCM, *Exocarpium Citri Grandis* (ECG) is known for its significant curative effects on pneumonic symptoms (China CftPoPsRo Committee for the Pharmacopoeia of People’s Republic of China, 2010). The most beneficial properties of ECG are strongly associated with its anti-oxidation (Tao and Liu, 2012), anti-bacterial (Jayaraman et al., 2012), and immunoregulation (Gyawali et al., 2012) properties. Composed of eight active ingredients, including ECG (a key constituent), the Juhongtanke oral solution (XiangXue Pharmaceutical Co. Ltd., Guangdong, China) is widely used in clinical practice in China. Although numerous pharmacological studies have revealed that Juhongtanke oral solution has promising therapeutic effects on CAP symptoms, their formal recognition as evidence-based is difficult because of the lack of high-quality randomized controlled trials (RCTs).

Inspired by previous research, this multicenter, open-label, parallel RCT aimed to evaluate the efficacy and safety of the Juhongtanke oral solution on pneumonic symptom alleviation in CAP patients.

## Material and methods

### Design and setting

This prospective, open-label, parallel-group RCT was conducted at the Department of Pulmonary Medicine of four tertiary hospitals in Guangdong Province, China. The participating clinical sites included Maoming People’s Hospital, Maoming Traditional Chinese Medicine Hospital, Gaozhou Hospital of Traditional Chinese Medicine, and the First People’s Hospital of Zhaoqing. The study protocol was designed following the CONSORT statement and approved by the ethics committee of the Maoming People’s Hospital, Maoming Traditional Chinese Medicine Hospital, Gaozhou Hospital of Traditional Chinese Medicine, and the First People’s Hospital of Zhaoqing (PJ2020MI-020-01, 20201011, yxllSJS2020018, and LC-2021-001, respectively). The protocol was built according to the Good Clinical Practice guidelines and performed following the Declaration of Helsinki. Written informed consent was obtained from all patients to participate in the study and publish the data. The registered information of protocol can be found on the China Clinical Trial Registry website (www.chictr.org/cn/No: ChiCTR2000039125).

### Blinding

This study was conducted as open-label with no blinding, both the patient and investigator knew about the medication intervened.

### Patients

Based on the study protocol, patients diagnosed with CAP were consecutively recruited from the participating centers between November 2020 and October 2021. Patients who met the following criteria were enrolled: 1) aged between 18 and 80 years; 2) diagnosed with CAP following the Guidelines For Primary Diagnosis and Treatment of Adult CAP (Chinese Medical Association, 2018) (Chinese Medical Association, 2019), and the Chinese criteria, which are also in line with the Diagnostic and Therapeutic Criteria of The American Thoracic Society (ATS)/Infectious Diseases Society of America (IDSA) (Metlay et al., 2019); 3) presented with CAP symptoms (e.g., cough, expectoration, or dyspnea); 4) can take oral medication; 5) patients or their legal surrogates who volunteered and signed a written informed consent form to participate in this study and publish the data. Patients were excluded from this study when they met the following criteria: 1) participating in or planning to participate in another clinical trial; 2) allergic to any constituents of the Juhongtanke oral solution; 3) cannot complete the Breathlessness, Cough and Sputum Scale (BCSS) and Visual Analogue Scale (VAS) independently; 4) suffer from severe diseases (e.g., malignancy, autoimmune disease, hepatic failure, or renal failure); 5) history of organ transplantation or splenectomy; 6) pregnancy.

Based on a computer-generated random number sequence, enrolled patients were allocated to the Juhongtanke oral solution or regular treatment arms at a ratio of 1:1, with a block size of 8, to minimize allocation bias. The randomization process was handled by the clinical research center of Maoming People’s Hospital. The randomization center verified the group when a patient enrollment was requested. The investigators did not reveal the randomization sequence, block numbers, and block sizes to preserve allocation concealment.

### Study intervention

Juhongtanke oral solution has been approved by the China Food and Drug Administration (approval code: Z44022180) and is available in China as an over-the-counter drug. ECG is the main active pharmaceutical ingredient of the Juhongtanke oral solution along with other ingredients, such as *Stemona japonica, Poria cocos, Pinellia, Glycyrrhiza uralensis fisch, Cynanchum glaucescens, Semen armeniacae amarum, and Schisandra chinensis.* The patients were randomized to receive routine treatment alone based on the Guidelines for Primary Diagnosis and Treatment of Adult CAP (Chinese Medical Association, 2018) (Chinese Medical Association, 2019) in the control group or the Juhongtanke oral solution (20 ml q8h) in the treatment group. The routine treatment advocated by the Chinese guideline under the recommendations of the ATS/IDSA was followed (Chinese Medical Association, 2019; Metlay et al., 2019). All participants received a 5-day treatment and an additional 3-day follow-up observation period.

### Data collection

Once the patients were enrolled, the following information was collected: 1) baseline demographics and clinical characteristics, including age, gender, body mass index (BMI), occupation, history of smoking, alcohol consumption, and vital signs such as body temperature, respiration, blood pressure, and heart rate; 2) diagnostics regarding principal diagnosis, comorbidities, allergic history, and the history of hypersensitivity to the study drug; 3) clinical efficacy of the study drug, which was assessed once per day with BCSS and VAS during the study. To interpret the procedure in greater detail, we performed BCSS and VAS assessments once patients were enrolled at the baseline period and 9:00 am daily before the first medication dose intake during the treatment (days 1–5). In addition, the length of hospital stay and in-hospital mortality were assessed. 4) Adverse events (AEs), including serious AEs, AEs leading to the withdrawal of the study drug, and AEs assessed related to study medication were also monitored and documented.

### Study outcomes

We applied BCSS and VAS as indicators to evaluate the treatment efficacy between treatment and control groups. The study’s primary outcome was defined as BCSS score measured on day 5 after the treatment. Secondary outcomes included BCSS score recorded from days 1–4, VAS score (cough and dyspnea) recorded from days 1–5, cumulative remission rate of BCSS and VAS in the 5-day protracted course, and the length of hospitalization and in-hospital mortality during the study. The remission in BCSS or VAS was defined as a reduction in score of at least 1 point from the baseline in the 5 days of treatment (Leidy et al., 2003; Huskisson, 2012). BCSS is a 12-point numerical rating scale ranging from 0 (no symptoms) to 12 (severely affected). It has been validated and is broadly used because it is a precise and simple tool for tracking the severity of respiratory symptoms and evaluating treatment efficacy in clinical trials of patients with infectious pneumonia (Leidy et al., 2003). The severity of cough and dyspnea was also assessed with VAS, which is also a widely used scale for pneumonic symptoms assessment (Huskisson, 2012). Using a 10-mm scale with verbal descriptors, a low end representing “no symptoms” and an upper end representing the worst symptoms were defined.

### Statistical analysis

Given our previous epidemiological and clinical investigation on CAP, the mean total score of BCSS in patients with CAP was expected to be 4.5 and 3.5 points before and after the administration of the Juhongtanke oral solution, respectively. The combined standard deviation (SD) of BCSS between the treatment and control groups was approximately 1.3. The superiority efficacy endpoints test was conducted at the *α* = 0.025 (one-sided) level with a statistical power of 0.8. Therefore, 120 subjects were planned to be included in each group to account for a potential withdrawal/dropped-out rate of 10% of the study population. The sample size was calculated using PASS 13.0 software.

For continuous data, the normality of distribution was assessed with the Shapiro-Wilk normality test. The continuous variables were presented as medians with interquartile range (IQR) or mean ± SDs based on their distribution. Categorical variables are presented as numbers and percentages. A student t-test was used to compare the means between the normally distributed variables, whereas the Wilcoxon-Mann-Whitney U-test was employed for the variables that were not normally distributed. Categorical variables were examined using Fisher’s exact test. Based on Kaplan-Meier method, the cumulative remission rate was compared with the log-rank test. The analysis dataset was selected based on the intention-to-treat set (ITTS), full analysis set (FAS), per-protocol set (PPS), and safety analysis set (SAS). Specifically, the primary analysis was performed on ITTS, whereas the efficacy analysis was performed on FAS and PPS. ITTS consisted of all randomized patients and FAS of ITTS patients who received ≥1 dose of the study drug. PPS included all randomized patients who completed the trial and did not have major protocol violations. The safety analysis was based on SAS, which included all patients who received ≥1 dose of the study drug. For the missing data in ITTS, FAS, and SAS, multiple imputations using the random forest algorithm from “MICE” R package were performed to develop imputed datasets (https://cran.r-project.org/web/packages/mice/). Statistical significance was considered *p*-values < 0.05. R programming language (version 4.0.2, https://www.rproject.org/) and IBM SPSS (version 25) were utilized for statistical analyses.

## Results

### Patients

**FIGURE 1:**
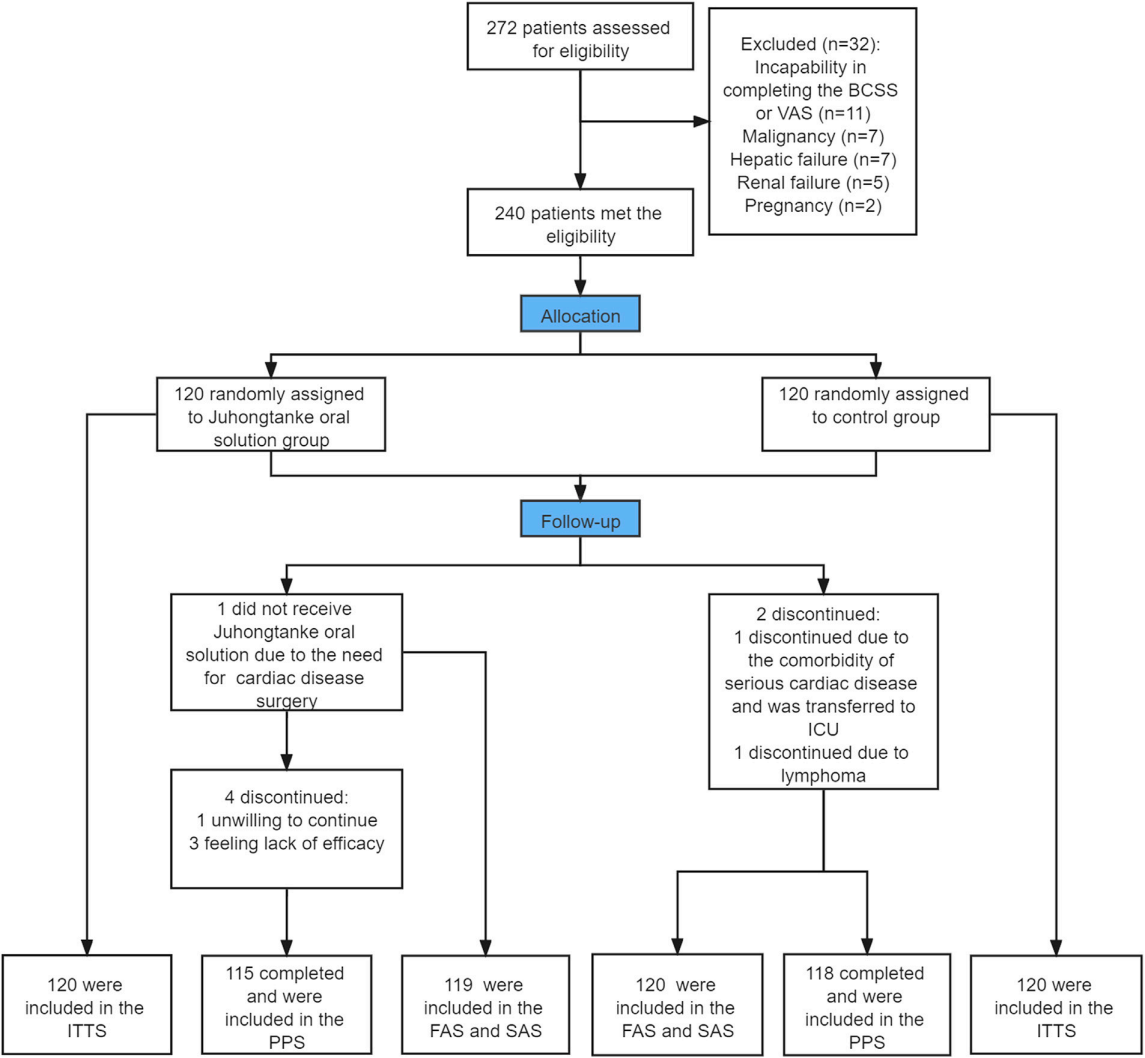
Study flow chart. ITTS, intention-to-treat set; FAS, full analysis set; PPS, per-protocol set; SAS, safety analysis set. BCSS, Breathlessness, Cough, and Sputum Scale; VAS, Visual Analogue Scale.

Of 272 patients assessed for eligibility, 240 patients were included in the study and randomly assigned to the treatment or control group at a ratio of 1:1. A total of 240 patients were included in ITTS (*n* =120 per group). One patient in the treatment group dropped out immediately following randomization and received no study drug, whereas all participants in the control group received at least one dose of the study treatment. Hence, 119 and 120 participants in the treatment and control groups were included in FAS and SAS, respectively. In addition, four participants dropped out from the treatment group; one due to unwillingness to continue and three due to feeling the lack of drug efficacy. Two patients dropped out from the control group due to the need for cardiac surgery and lymphoma diagnosis. Therefore, 115 patients in the treatment group and 118 patients in the control group were included in PPS (Figure 1). No participants withdrew from the trial due to medication side effects.

### Baseline characteristics

As indicated in Table 1, both groups tended to be middle-aged. No statistically significant differences were observed between the two groups regarding gender, BMI, vital signs, smoking history, drinking history, comorbidity, routine treatment of CAP symptoms, and antibiotics use.

**TABLE 1:** Baseline demographic and clinical characteristics of the patients from ITTS.

Variables	Description	Treatment group (*n* = 120)	Control group(*n* = 120)	*p-*value
Age (year, mean (SD))		56.39 ± 13.60	55.01 ± 14.62	0.451
Gender (n, %)	Male	61 (50.83)	65 (54.17)	0.605
	Female	59 (49.17)	55 (45.83)	
Body mass index (kg/m^2^, mean (SD))		22.56 ± 4.20	22.59 ± 3.30	0.956
Body temperature (°C, mean (SD))		36.68 ± 0.66	36.72 ± 0.67	0.635
Heart rate (bpm, mean (SD))		87.34 ± 13.75	85.76 ± 13.61	0.373
Respiratory rate (bpm, mean (SD))		20.86 ± 1.27	21.00 ± 1.25	0.386
Systolic blood pressure (mmHg, mean (SD))		125.44 ± 17.61	128.45 ± 17.71	0.190
Diastolic blood pressure (mmHg, mean (SD))		78.68 ± 12.90	80.40 ± 10.89	0.266
Educational attainment (n, %)				0.553
	Primary school	58 (48.33)	50 (41.67)	
	Middle school	26 (21.67)	29 (24.17)	
	High school	27 (22.50)	27 (22.50)	
	Polytechnic school	6 (5.00)	6 (5.00)	
	College	3 (2.50)	8 (6.67)	
Smoking history (n, %)				0.778
	No	85 (70.83)	83 (69.17)	
	Yes	35 (29.17)	37 (30.83)	
Drinking history (n, %)				0.392
	No	115 (95.83)	112 (93.33)	
	Yes	5 (4.17)	8 (6.67)	
Allergic history (n, %)				0.250
	No	118 (98.33)	115 (95.83)	
	Yes	2 (1.67)	5 (4.17)	
Comorbidity (n, %)				0.750
	None	70 (58.33)	76 (63.33)	
	COPD	9 (7.50)	9 (7.50)	
	Bronchiectasis	8 (6.67)	5 (4.17)	
	Asthma	4 (3.33)	3 (2.50)	
	Pleuritis	1 (0.83)	2 (1.67)	
	Chronic Bronchitis	3 (2.50)	7 (5.83)	
	CVD	16 (13.33)	10 (8.33)	
	DM	2 (1.67)	2 (1.67)	
	Liver disease	6 (5.00)	3 (2.50)	
	Kidney disease	1 (0.83)	2 (1.67)	
	Lymphoma	0(0.00)	1 (0.83)^a^	
Antitussive treatment (n, %)				0.471
	Compound-methoxyphenamine Capsule	47(39.17)	42(35.00)	
	Dextromethorphan	49(40.83)	55(45.83)	
	Others	4(3.33)	1(0.83)	
	Not received	20(16.67)	22(18.33)	
Expectorant treatment (n, %)				0.541
	Eucalyptol	7(5.83)	5(4.17)	
	Ambroxol	32(26.67)	38(31.67)	
	Bromhexine	22(18.33)	20(16.67)	
	Acetylcysteine	13(10.83)	21(17.50)	
	Others	12(10.00)	9(7.50)	
	Not received	34(28.33)	27(22.50)	
Anti-dyspnea treatment (n, %)				0.698
	Theophylline	38(31.67)	35(29.17)	
	Ipratropium bromide	15(12.500)	19(15.83)	
	Terbutaline	14(11.67)	15(12.50)	
	Budesonide	16(13.33)	12(10.00)	
	Others	1(0.83)	4(3.33)	
	Not received	36(30.00)	35(29.17)	
Antibiotic treatment (n, %)				0.738
	Cephalosporin	72(60.00)	80(66.67)	
	β-lactam	18(15.00)	11(9.17)	
	Quinolone	24(20.00)	21(17.50)	
	Aminoglycoside	1(0.83)	2(1.67)	
	Macrolide	4(3.333)	5(4.167)	
	Carbapenem	1(0.83)	1(0.83)	

Data presented as mean (standard deviation, SD) or (n, %). ITTS, intention-to-treat set; COPD, chronic obstructive pulmonary disease; CVD, cardiovascular disease; DM, diabetes mellitus;

^a^ The patient dropped out from the study in the control group.

### Primary outcome

**FIGURE 2:**
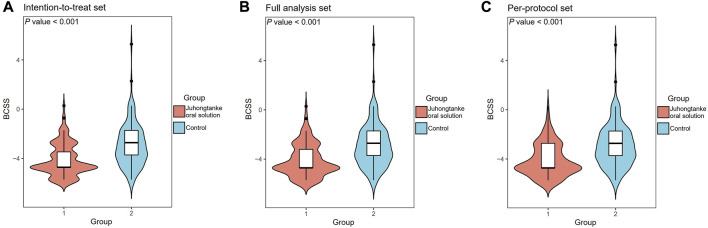
Comparison of BCSS on day-5 of the study course between the Juhongtanke oral solution and control groups in the ITTS **(A)**, FAS **(B)**, and PPS **(C)**.

For the primary outcome in the ITTS, the mean difference in BCSS is a median of –4 points [interquartile range (IQR) (–6.00, –3.00)] change from baseline in the treatment group, compared to the –3 points [IQR (–4.00, –2.00)] median in the control group. The mean difference in BCSS between the treatment and control groups was a median of 1 point [IQR (1.00, 2.00)] (*p* < 0.001). Similarly, in FAS and PPS populations, the mean difference in BCSS between treatment and control groups was a median of 1 point [ IQR (1.00, 2.00)] (*p* < 0.001) (Table 2 and Figure 2).

**TABLE 2:** Comparison of the primary and secondary endpoints.

Variables	Intention-to-treat set				Full analysis set				Per-protocol set			
	Treatment group *n* =120	Control grou *n* =120	Difference in change (95% CI)<	*p*-value	Treatment group *n* =119	Control group *n* =120	Difference in change (95% CI)	*p*-value	Treatment group *n* =115	Control group *n* =118	Difference in change (95% CI)	*p*-value

BCSS, baseline (points, median (IQR))	6.00 (5.00, 7.00)	6.00 (4.00, 7.00)	0.00 (−1.00,0.00)	0.488	6.00 (5.00, 7.00)	6.00 (4.00, 7.00)	0.00 (−1.00,0.00)	0.520	6.00 (5.00,7.00)	6.00 (4.00, 7.00)	0.00 (−1.00,0.00)	0.445
^a^cBCSS-1 (points, median (IQR))	−1.00 (−1.00, 0.00)	0.00 (−1.00, 0.00)	0.00 (0.00,0.00)	0.019	−1.00 (−1.00, 0.00)	0.00 (−1.00, 0.00)	0.00 (0.00,0.00)	0.021	−1.00 (−1.00, 0.00)	0.00 (−1.00, 0.00)	0.00 (0.00,0.00)	0.027
cBCSS-2 (points, median (IQR))	−2.00 (−3.00, −1.00)	−1.00 (−2.00, −1.00)	1.00 (0.00,1.00)	<0.001	−2.00 (−3.00, −1.00)	−1.00 (−2.00, −1.00)	1.00 (0.00,1.00)	<0.001	−2.00 (−3.00, −1.00)	−1.00 (−2.00, −1.00)	1.00 (0.00,1.00)	<0.001
cBCSS-3 (points, median (IQR))	−3.00 (−4.00, −2.00)	−2.00 (−3.00, −1.00)	1.00 (1.00,1.00)	<0.001	−3.00 (−4.00, −2.00)	−2.00 (−3.00, −1.00)	1.00 (1.00,1.00)	<0.001	−3.00 (−4.00, −2.00)	−2.00 (−3.00, −1.00)	1.00 (1.00,1.00)	<0.001
cBCSS-4 (points, median (IQR))	−4.00 (−5.00, -2.00)	−3.00 (−3.00, −2.00)	1.00 (1.00,2.00)	<0.001	−4.00 (−5.00, −2.00)	−3.00 (−3.00, −2.00)	1.00 (1.00,1.00)	<0.001	−4.00 (−5.00, −2.00)	−3.00 (−3.00, −2.00)	1.00 (1.00,	<0.001
cBCSS-5 (points, median (IQR))	−4.00 (−6.00, −3.00)	−3.00 (−4.00, −2.00)	1.00 (1.00,2.00)	<0.001	−4.00 (−6.00, −3.00)	−3.00 (−4.00, −2.00)	1.00 (1.00,2.00)	<0.001	−4.00 (−6.00, −3.00)	−3.00 (−4.00, −2.00)	1.00 (1.00,2.00)	<0.001
VASD, baseline (points, median (IQR))	3.50 (1.00, 6.48)	4.00 (0.00, 6.23)	0.00 (−0.70, 0.90)	0.956	3.50 (1.00, 6.50)	4.00 (1.00, 6.23)	0.00 (−0.60, 1.00)	0.915	3.50 (1.00,6.50)	4.00 (1.00, 6.33)	0.00 (-0.90, 0.80)	0.956
cVASD-1 (points, median (IQR))	**-**0.10 (−1.00, 0.00)	0.00 (−0.28, 0.00)	0.00 (0.00,0.50)	<0.001	0.00 (−1.00, 0.00)	0.00 (−0.28, 0.00)	0.00 (0.00,0.40)	<0.001	−0.20 (−1.00, 0.00)	0.00 (−0.23, 0.00)	0.00 (0.00,0.50)	<0.001
cVASD-2 (points, median (IQR))	−1.00 (−2.38, 0.00)	−0.60 (−1.00, 0.00)	0.50 (0.00,1.00)	<0.001	-1.00 (−2.00, 0.00)	-0.60 (−1.00, 0.00)	0.40 (0.00,1.00)	<0.001	−1.00 (−2.00, 0.00)	−0.60 (−1.00, 0.00)	0.50 (0.00,1.00)	<0.001
cVASD-3 (points, median (IQR))	−2.00 (−3.50, 0.00)	−1.00 (−1.85, 0.00)	1.00 (0.00,1.00)	<0.001	−2.00 (−3.50, 0.00)	−1.00 (−1.85, 0.00)	0.90 (0.00,1.00)	<0.001	−2.00 (−3.50, 0.00)	−1.00 (−1.75, 0.00)	1.00 (0.00,1.00)	<0.001
cVASD-4 (points, median (IQR))	−2.55 (−4.38, −1.00)	−1.45 (−2.00, −0.50)	1.00 (0.40,1.50)	<0.001	−2.50 (−4.00, −1.00)	−1.45 (−2.00, −0.50)	1.00 (0.30,1.50)	<0.001	−2.60 (−4.00, −1.00)	−1.45 (−2.00, −0.43)	1.00 (0.50,1.50)	<0.001
cVASD-5 (points, median (IQR))	−3.00 (−5.00, −1.00)	−2.00 (−2.50, −1.00)	1.00 (0.50,2.00)	<0.001	−3.00 (−5.00, −1.00)	−2.00 (−2.50, −1.00)	1.00 (0.50,2.00)	<0.001	−3.00 (−5.00, −1.00)	−2.00 (−2.50, −1.00)	1.00 (0.50,2.00)	<0.001
VASC, baseline (points, median (IQR))	6.00 (4.00, 8.00)	6.10 (4.00, 7.50)	0.00 (−0.60, 0.20)	0.708	6.00 (4.00, 8.00)	6.10 (4.00, 7.50)	0.00 (-0.80, 0.00)	0.661	6.00 (4.00,8.00)	6.10 (4.00, 7.50)	0.00 (−1.00, 0.00)	0.532
cVASC-1 (points, median (IQR))	−0.90 (−1.00, 0.00)	−0.20 (−0.98, 0.00)	0.30 (0.00,0.50)	<0.001	−1.00 (−1.00, 0.00)	−0.20 (−0.98, 0.00)	0.30 (0.00,0.50)	<0.001	−1.00 (−1.00, 0.00)	−0.20 (−1.00, 0.00)	0.30 (0.00,0.50)	<0.001
cVASC-2 (points, median (IQR))	−1.60 (−3.00, −1.00)	−1.00 (−1.00, 0.00)	1.00 (0.50, 1.00)	<0.001	−1.50 (−3.00, −1.00)	−1.00 (−1.00, 0.00)	1.00 (0.50, 1.00)	<0.001	−2.00 (−3.00, −1.00)	−1.00 (−1.00, −0.50)	1.00 (0.50, 1.00)	<0.001
cVASC-3 (points, median (IQR))	−2.55 (−4.00, −1.00)	−1.35 (−2.00, −−1.00)	1.00 (1.00,1.50)	<0.001	−2.60 (−4.00, −1.00)	−1.35 (−2.00, −1.00)	1.00 (1.00,1.50)	<0.001	−3.00 (−4.00, −1.00))	−1.35 (−2.00, −1.00)	1.00 (1.00,1.70)	<0.001
cVASC-4 (points, median (IQR))	−3.35 (−5.00, −2.00)	−2.00 (−2.78, −1.00)	1.50 (1.00,2.00)	<0.001	−3.50 (−5.00, −2.00)	−2.00 (−2.78, −1.00)	1.50 (1.00,2.00)	<0.001	−4.00 (−5.00, −2.00)	−2.00 (−2.78, −1.00)	1.50 (1.00,2.00)	<0.001
cVASC-5 (points, median (IQR))	−4.70 (−6.00, −3.00)	−2.4 (−3.15, −1.53)	2.00 (1.40,2.50)	<0.001	−4.80 (−6.00, −3.00)	−2.4 (−3.15, −1.53)	2.00 (1.50,2.50)	<0.001	−4.90 (−6.20, −3.00)	−2.4 (−3.28, −1.50)	2.00 (1.50,2.50)	<0.001
BCSS remission rate in the 5-day treatment course (n, %)	117 (99.50)	107 (89.17)	8.33 (1.33,15.88)	0.010	116 (97.48)	107 (89.17)	8.31 (1.28,15.86)	0.010	112 (99.39)	105 (88.98)	8.41 (1.20,16.08)	0.011
VASD remission rate in the 5-day treatment course (n, %)	106 (88.33)	75 (62.50)	25.83 (14.5036.39)	<0.001	105 (88.24)	75 (62.50)	25.74 (14.37,36.31)	<0.001	101 (87.82)	72 (61.01)	26.81 (15.16,37.55)	<0.001
VASC remission rate in the 5-day treatment course (n, %)	118 (98.33)	90 (75.00)	23.33 (14.62, 32.32)	<0.001	117 (98.32)	90 (75.00)	23.32 (14.59, 32.31)	<0.001	113 (98.26)	88 (74.58)	23.68 (14.77, 32.79)	<0.001
BCSS remission time (days, median (IQR))	2.00 (1.00,3.00)	2.00 (1.00,4.00)	0.00 (0.00,1.00)	0.074	2.00 (1.00,3.00)	2.00 (1.00,4.00)	0.00 (0.00,<1.00)	0.072	2.00 (1.00,3.00)	2.00 (1.00,4.00)	0.00 (0.00,1.00)	0.068
VASD remission time (days, median (IQR))	1.00 (1.00,3.00)	3.00 (1.00,<5.00)	0.00 (0.00,1.00)	0.002	1.00 (1.00,3.00)	3.00 (1.00,5.00)	0.00 (0.00,1.00)	0.002	2.00 (1.00,3.00)	3.00 (1.00,5.00)	0.00 (0.00,1.00)	0.003
VASC remission time (days, median (IQR))	2.00 (1.003.00)	4.00 (1.00,5.00)	1.00 (0.00,1.00)	0.001	2.00 (1.00,3.00)	4.00 (1.00,5.00)	1.00 (0.00,1.00)	0.001	2.00 (1.00,3.00)	4.00 (1.00,5.00)	1.00 (0.00,1.00)	0.002
Length of hospital stays (days, median (IQR))	7.00 (6.00, 9.00)	7.00 (6.00, 9.00)	0.00 (−1.00,1.00)	0.871	7.00 (6.00, 9.00)	7.00 (6.00, 9.00)	0.00 (−1.001.00)	0.841	7.00 (6.00, 9.00)	7.00 (6.00, 9.00)	0.00 (−1.00	0.806
Death (n, %)	0.00 (0.00)	0.00 (0.00)	NA	NA	0.00 (0.00)	0.00 (0.00)	NA	NA	0.00 (0.00)	0.00 (0.00)	NA	NA

Data presented as median (interquartile range, IQR) or (n, %). CI, confidence interval; NA, not applicable; BCSS, breathlessness, Cough and Sputum Scale; VAS, visual analogue scale; VASD, VAS of dyspnea; VASC, VAS of cough.

^a^ cBCCS-1, BCSS, score change from baseline on day-1, and so on.

### Secondary outcomes

**FIGURE 3:**
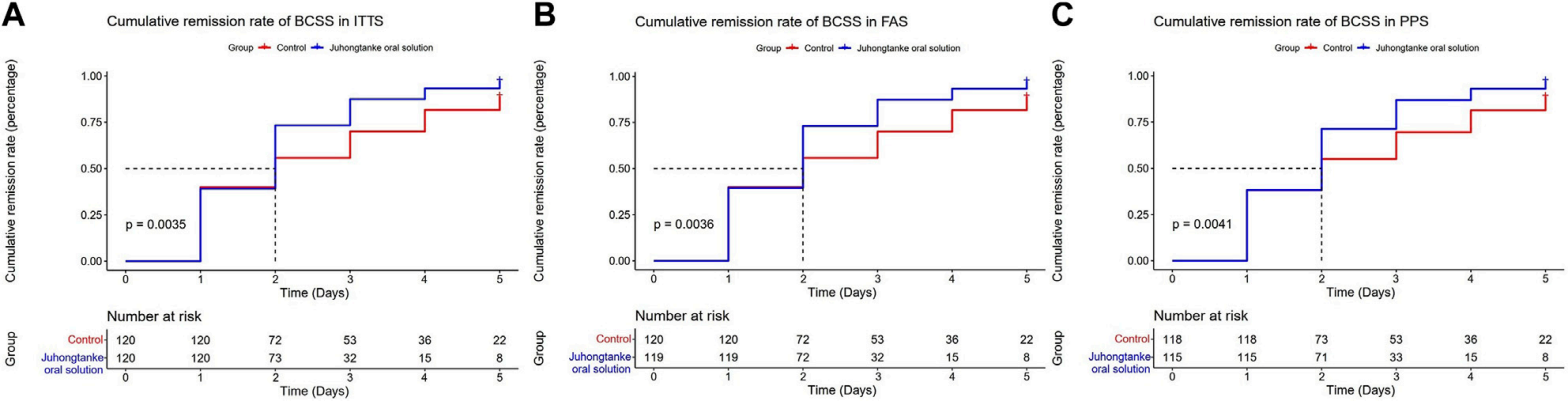
Cumulative remission rate of BCSS between the Juhongtanke oral solution and control groups in the ITTS **(A)**, FAS **(B)**, and PPS **(C)**.

**FIGURE 4:**
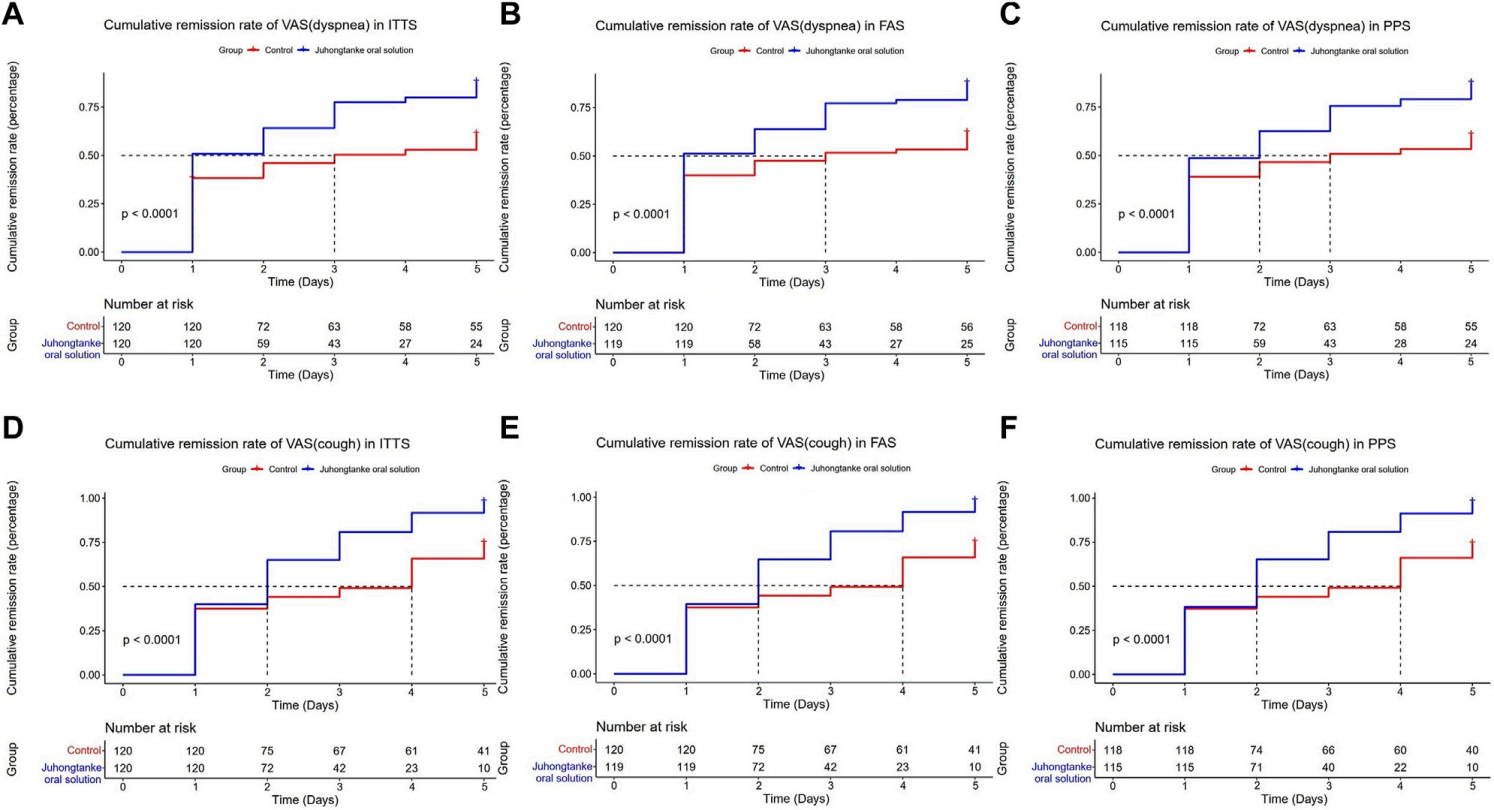
Cumulative remission rate of VAS (dyspnea and cough) between the Juhongtanke oral solution and control groups in the ITTS **(A,D)**, FAS **(B,E)**, and PPS **(C,F)**.

As presented in Table 2, BCSS scores between groups were statistically significant on days 1, 2, 3, and 4 for ITTS, FAS, and PPS analyses. For ITTS, the mean differences between the groups through day 1–4 were a median 0 point [95% CI (0.00, 0.00)] (*p* = 0.019), median 1 point [95% CI (0.00, 1.00)] (*p* < 0.001), median 1 point [95% CI (1.00, 1.00)] (*p* < 0.001), and median 1 point [95% CI (1.00, 2.00)] (*p* < 0.001), respectively. Since day 1, a significant difference in the item of cough in VAS was observed between the treatment and control groups in the ITTS, FAS, and PPS analyses. For ITTS, the mean differences between the groups through day 1–5 were a median of 0.3 point [95% CI (0.00, 0.50)] (*p* < 0.001), median 1 point [95% CI (0.50, 1.00)] (*p* < 0.001), median 1 point [95% CI (1.00, 1.50)] (*p* < 0.001), median 1.5 point [95% CI (1.00, 2.00)] (*p* < 0.001), and median 2 point [95% CI (1.40, 2.50)] (*p* < 0.001), respectively. For the item of dyspnea in VAS, significant differences also appeared on day 1 in ITTS, FAS, and PPS. For ITTS, the mean differences between the groups through days 1–5 were a median of 0 point [95% CI (0.00, 0.50)] (*p* < 0.001), median 0.5 point [95% CI (0.00, 1.00)] (*p* < 0.001), median 1 point [95% CI (0.00, 1.00)] (*p* < 0.001), median 1 point [95% CI (0.40, 1.50)] (*p* < 0.001), and median 1 point [95% CI (0.50, 2.00)] (*p* < 0.001), respectively. Figures 3, 4 display the cumulative remission rate of BCSS and VAS scores, demonstrating that the treatment group had more favorable outcomes in symptom alleviation than the control group in ITTS, FAS, and PPS. For ITTS, the remission rate of BCSS was 99.50% in the treatment group vs. 89.17% in the control group (*p* = 0.010). The cough remission rate was 98.33% in the treatment group vs. 75.00% in the control group (*p* < 0.001), while the dyspnea remission rate was 88.33% in the treatment group vs. 62.50% in the control group (*p* < 0.001). In addition, no significant differences regarding the length of hospital stay were observed among ITTS, FAS, and PPS (with a median of 7 days in the triple sets, with all *p* < 0.001) groups.

### Safety

According to the results of SAS, several AEs were recorded during the study. Dry mouth was the most common complication for the group receiving Juhongtanke oral solution compared to the routine treatments, and it was reported in two and one individuals in treatment and control groups, respectively. One patient experienced mild symptoms of urticaria after taking the Juhongtanke oral solution. His condition soon improved after conservative symptomatic treatment. No patient withdrew from the study because of local AEs, and no treatment-related deaths occurred during our study (Table 3).

**TABLE 3:** Summary of AEs in SAS.

Variables	Description	Treatment group *n* = 119	Control group *n* = 120	*p*-value
Fatigue (n, %)	No	119 (100.00)	120 (100.00)	NA
Headache (n, %)	No	119 (100.00)	120 (100.00)	NA
Chest distress (n, %)	No	119 (100.00)	120 (100.00)	NA
Palpitation (n, %)	No	119 (100.00)	120 (100.00)	NA
Elevated blood pressure (n, %)	No	119 (100.00)	120 (100.00)	NA
Upset stomach (n, %)	No	119 (100.00)	120 (100.00)	NA
Dizziness (n, %)				1.000
	No	119 (100.00)	119 (99.17)	
	Yes	0 (0.00)	1 (0.83)	
Dry mouth (n, %)				0.994
	No	117 (98.32)	119 (99.17)	
	Yes	2 (1.68)	1 (0.83)	
Urticaria (n, %)				0.498
	No	118 (99.16)	120 (100.00)	
	Yes	1 (0.84)	0 (0.00)	

Data presented as (n, %). AEs, adverse events; SAS, safety analysis set; NA, not applicable.

## Discussion

To the best of our knowledge, this is the first clinical trial that applied a rigorous and widely adopted approach to assess the efficacy and safety of Juhongtanke oral solution for CAP adjuvant management. Overall, our study indicated that combining Juhongtanke oral solution and routine treatment can better improve BCSS and VAS scores in patients with CAP compared with the control. Besides, no noticeable side effects relevant to the study drug were observed.

In clinical applications, the Juhongtanke oral solution is widely used as an anti-tussive, expectorant, and anti-dyspnea TCM for managing chronic bronchitis (Jiang et al., 2014). Unlike synthetic chemical drugs, TCMs and their preparations are popular for improving therapeutic effectiveness through the synergistic effects of multiple active components. Animal studies revealed that multiple bio-activities of Juhongtanke oral solution are mainly exerted by the key component of ECG and other dominant ingredients such as *Poria cocos, Pinellia,* and *Schisandra chinensis* (China CftPoPsRo Committee for the Pharmacopoeia of People’s Republic of China, 2010; Gao et al., 2011; Liu et al., 2011). ECG can inhibit the responses of rapidly adapting receptors, which results in decreased bronchial hyperreactivity and mucus hypersecretion (Liu et al., 2011). Furthermore, ECG can exert anti-inflammatory, immunomodulatory, and anti-oxidant effects through multiple pathways (Jayaraman et al., 2012; Jiang et al., 2014; Yang et al., 2018). Similar to ECG, *Poria cocos* can relieve cough as an expectorant by targeting nuclear factor-κB, mitogen-activated protein kinase, and TGFRII signaling pathway (Hu et al., 2021b; Gui et al., 2021; Lu et al., 2021), whereas *Pinellia* and *Schisandra chinensis* perform similar roles in pneumonia by participating in various regulatory pathways in animal models (Su et al., 2016; Yang et al., 2021).

Based on animal disease models, Juhongtanke oral solution or its components have demonstrated a good therapeutic effect on pneumonic symptom alleviation; however, the scientific values regarding the prediction of CAP patients remain limited. To further validate the evidence of Juhongtanke oral solution on CAP management, we retrieved clinical trials published in Chinese based on the Wanfang (http://www.wanfangdata.com.cn) and China National Knowledge Infrastructure (http://www.cnki.net/) databases between 1980 and November 2021. The results were dis-satisfactory because the data for assessing the Juhongtanke oral solution in patients with CAP were inadequate, and most of the evidence was notably weak to direct clinical practice. The strength of previous studies was possibly limited to their retrospective design, misleading patient stratification, and limited by the lack of a widely accepted tool for pneumonic symptom assessment (Zhang, 2017; Cui, 2019; Yang et al., 2019). Considering the shortage of evidence to either support or refute the routine use of Juhongtanke oral solution by patients with CAP, an appropriate clinical trial must be established.

Unlike the studies outlined above, the strengths of our research lie not only in its multi-center, prospective, randomized design but also in the assessment of pneumonic symptoms. Either BCSS or VAS used in this study has been widely applied and validated in evaluating pneumonia symptoms. In particular, combining Juhongtanke oral solution and routine treatment was superior to the recommended routine treatment prescribed by ATS/IDSA and Chinese guidelines in BCSS and VAS assessments (Cao et al., 2018; Chinese Medical Association, 2019; Metlay et al., 2019). Statistical differences in BCSS were observed since day 1 of the study, despite the primary endpoint for pneumonic symptom assessment being set to day 5 based on our previous experience (Figure 2). Compared with the control, a significant reduction in VAS scores (cough and dyspnea) was also observed from day 1 in all analysis sets (Table 2). Therefore, the present findings suggest that the Juhongtanke oral solution has a remarkable therapeutic effect on pneumonic symptom alleviation. However, our study did not observe improvement in the length of hospital days associated with Juhongtanke oral solution. The most likely reason is our strict adherence to the discharge criteria determined by the current clinical guidelines (a total recovery from the infection) (Chinese Medical Association, 2019; Metlay et al., 2019), although the pneumonic symptoms were eased to a large extent after receiving the study drug. On the other hand, given that the primary objective of the study was targeted at drug assessment during pneumonia symptom alleviation, other potential reasons can explain the lack of difference in the length of hospital days or in-hospital mortality between the two groups. Future studies focusing on these additional concerns are warranted to evaluate the clinical applications of Juhongtanke oral solution in CAP management.

Regarding drug safety, the occurrence of adverse drug reactions among patients during the 8-day observation was relatively low and showed no statistical discrepancy compared with the control group (Table 3). Furthermore, no in-hospital mortality was observed in our study (Table 2). These findings were based on a regular therapeutic dose recommended by the manufacturer’s instructions. Given that Juhongtanke oral solution has multi-therapeutic effects on treating respiratory diseases, no optimal dose exists for patients with CAP. However, based on our observations, we believe that the current dose of Juhongtanke oral solution is safe and effective for this population.

Although this study provides several insights into the clinical practice of Juhongtanke oral solution in CAP patients, the limitations in terms of controlled drug selection, application of blinding, and safety observation need to be revealed. First, given the lack of standard medical therapy for pneumonic symptoms with the current clinical guidelines, designating a specific drug for the control group would be a selection bias risk. Second, the present study was not double-blinded because of variations in appearance, odors, tastes, and the usage of medications between treatment and control groups. Third, outcome assessments were based on self-reporting, which could introduce subjective bias. Consequently, several inherent weaknesses cannot be completely ruled out. To minimize these potential sources of bias, we adequately applied rigorous randomization and allocation concealment methods to the enrolled patients. To ensure that the scale rating was as accurate as possible, all investigators were professionally trained and well-versed in GCP; hence, face-to-face interviews were conducted by experienced investigators. No serious AEs were observed in this study, which may be due to the limited duration of the trial. Accordingly, further studies with larger sample sizes and longer observation periods are required to confirm these results.

## Conclusion

Our study suggests that Juhongtanke oral solution may be beneficial for pneumonic symptom alleviation and thus might be valuable for the clinical management of CAP patients.

## Data Availability

The datasets used and/or analyzed during the current study are available from the corresponding author on reasonable request.

## Abbreviations

ATS, American thoracic society; AE, adverse event; BMI, body mass index; BCSS, breathlessness, cough, and sputum scale; CAP, community-acquired pneumonia; CI, confidence interval; COPD, chronic obstructive pulmonary disease; CVD, cardiovascular disease; DM, diabetes mellitus; ECG, Exocarpium citri grandis; FAS, full analysis set; IDSA, infectious diseases society of America; IQR, Interquartile range; ITTS, Intention-to-treat set; NA, not applicable; PPS, per-protocol set; RCT, randomized controlled trial; VAS, Visual analogue scale; VASD, VAS of dyspnea; VASC, VAS of cough; TCM, traditional Chinese medicine.
